# The gut microbiome: a core regulator of metabolism

**DOI:** 10.1530/JOE-22-0111

**Published:** 2023-01-19

**Authors:** Shiho Fujisaka, Yoshiyuki Watanabe, Kazuyuki Tobe

**Affiliations:** 1 First Department of Internal Medicine, Faculty of Medicine, University of Toyama, Sugitani, Toyama, Japan

**Keywords:** obesity, diabetes, metabolism

## Abstract

The human body is inhabited by numerous bacteria, fungi, and viruses, and each part has a unique microbial community structure. The gastrointestinal tract harbors approximately 100 trillion strains comprising more than 1000 bacterial species that maintain symbiotic relationships with the host. The gut microbiota consists mainly of the phyla Firmicutes, Bacteroidetes, Proteobacteria, and Actinobacteria. Of these, Firmicutes and Bacteroidetes constitute 70–90% of the total abundance. Gut microbiota utilize nutrients ingested by the host, interact with other bacterial species, and help maintain healthy homeostasis in the host. In recent years, it has become increasingly clear that a breakdown of the microbial structure and its functions, known as dysbiosis, is associated with the development of allergies, autoimmune diseases, cancers, and arteriosclerosis, among others. Metabolic diseases, such as obesity and diabetes, also have a causal relationship with dysbiosis. The present review provides a brief overview of the general roles of the gut microbiota and their relationship with metabolic disorders.

## Physiological role of the gut microbiota

### Nutrient metabolism and absorption

#### Degradation of indigestible polysaccharides

**Figure 1:**
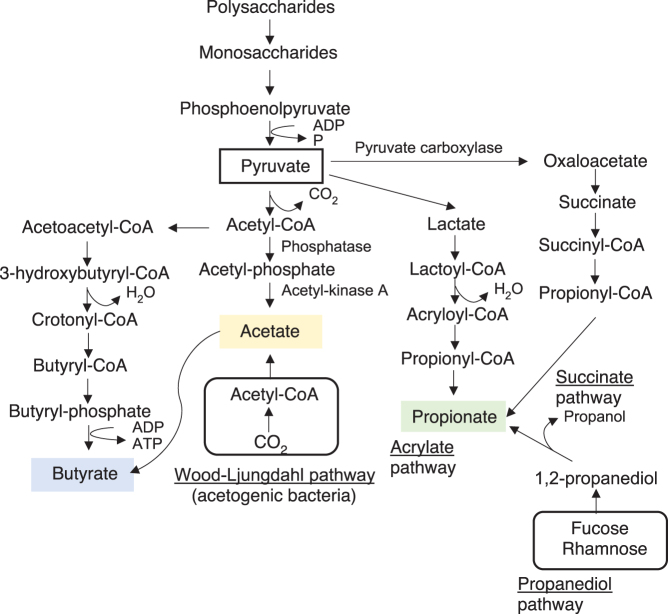
Short-chain fatty acid (SCFA) biosynthesis pathways.

Short-chain fatty acids (SCFAs) are metabolites generated from the fermentation of insoluble dietary fiber and indigestible polysaccharides by the gut microbiota. Linear monovalent carboxylic acids with fewer than six carbons, such as acetate (C2), propionate (C3), and butyrate (C4), are the most abundant SCFAs, with average ratios of ~60%:20%:20%. Total intestinal SCFA concentrations can reach 100 mM (Cummings *et al.* 1987). Butyrate is the main nutrient source for colonocytes. Acetate has the highest intestinal concentration of all SCFAs. It enters the liver via the portal vein, undergoes oxidation, and is used mainly by hepatocytes. Propionate participates in hepatic gluconeogenesis. These SCFAs maintain the barrier function of the intestinal tract, are bioactive substances in energy metabolism (Morrison and Preston 2016), regulate immunocyte development, and are anti-inflammatory (Louis *et al.* 2014, Richards *et al.* 2016). Various pathways are involved in SCFA synthesis (Koh *et al.* 2016). Acetate is synthesized from pyruvate during glycolysis and by acetogenic bacteria via the Wood–Ljungdahl pathway, which produces acetyl-CoA from CO_2_. Acetoacetyl-CoA is converted to butyryl-CoA, which, in turn, is transformed into butyrate. Major butyrate-producing bacterial taxa include the Ruminococcaceae, Lachnospiraceae, Erysipelotrichaceae, and Clostridiaceae of the Firmicutes phylum (Barcenilla *et al.* 2000, Louis *et al.* 2004). *Clostridium* spp. (such as *C. butyricum*) and *Butyrivibrio* spp. (such as *B. fibrisolvens*) also produce butyrate. *Acetobacter* spp. and *Gluconobacter* spp. produce acetate (Louis *et al.* 2014, Knip & Siljander 2016, Koh *et al.* 2016). SCFAs are interconverted, and 24% of all acetate is converted to butyrate (Boets *et al.* 2017). Pathways involved in propionate synthesis include the acrylate pathway mediated by lactate metabolism, the succinate pathway mediated by succinate, and the propanediol pathway utilizing deoxy sugars such as fucose and rhamnose (Fig. 1) (Louis & Flint 2017). *Akkermansia muciniphila* and other species are propionate-producing, mucin-degrading bacteria (Derrien *et al.* 2004). SCFAs are important not only for energy and glucose metabolism, inflammation, and immune function regulation (Koh *et al.* 2016) but also for intestinal environment maintenance. For example, butyrate is important for maintaining an anaerobic environment in the intestinal tract and activating PPARγ by promoting mitochondrial β-oxidation in intestinal epithelial cells (IECs), maintaining hypoxia for epithelial cells and inhibiting oxygen transfer into the intestinal lumen (Rivera-Chavez *et al.* 2016, Byndloss *et al.* 2017). Acetate and propionate produced in the intestine also play an important role in intestinal homeostasis. They induce colonic Treg to protect against experimentally induced colitis (Smith *et al.* 2013). Another group reported that acetate suppresses intestinal inflammation via G protein-coupled receptor (GPR) 43 signaling expressed on neutrophils (Maslowski *et al.* 2009). It also enhances intestinal barrier function and contributes to defense against pathogens (Fukuda *et al.* 2011).

#### Bile acids and lipid metabolism

**Figure 2:**
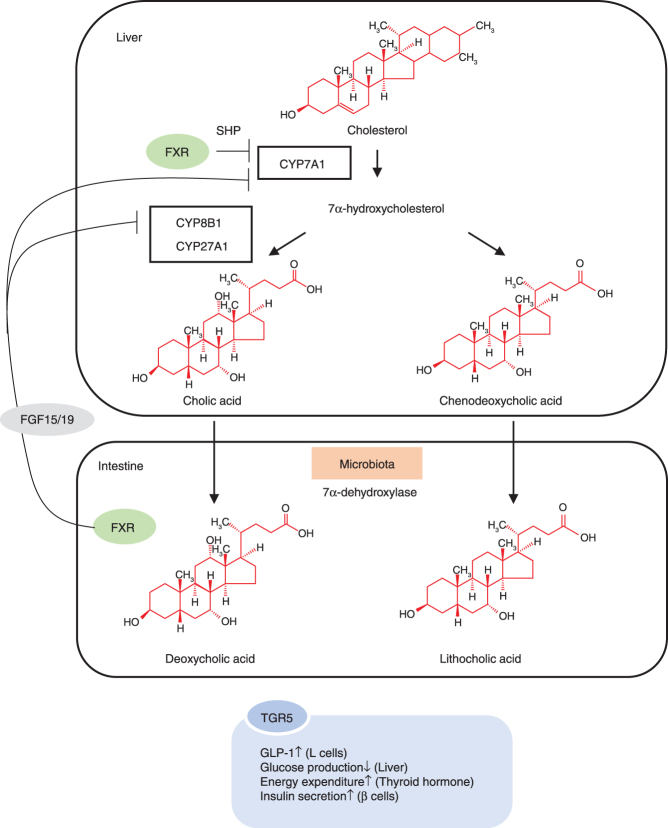
Bile acid metabolism. The microbiota dehydroxylate primary bile acids, such as cholic acid and chenodeoxycholic acid, to secondary bile acids with 7a-dehydroxylase. Activation of the bile acid receptor FXR suppresses the expression of CYP7A1, a bile acid rate-limiting enzyme, via the induction of small heterodimer partner (SHP) expression in the liver to control bile acid synthesis. In addition, FXR activation in the intestine suppresses CYP7A1 and CYP8B1 expression via the induction of FGF15/19 expression.

Secondary bile acids are major bacterial metabolites affecting host physiology. Hepatocytes produce cholic acid and chenodeoxycholic acid from cholesterol. These lipid-soluble bile acids are conjugated to glycine or taurine to form water-soluble bile salts and are stored in the gallbladder and released into the intestine during digestion. Gut bacteria dehydroxylate primary bile salts into secondary bile salts. These bile acids are actively resorbed along the proximal and distal ileum into the hepatic portal circulation. Bacteria also deconjugate certain primary and secondary conjugated bile salts into lipid-soluble bile acids that are passively absorbed into the hepatic portal circulation. Approximately 95% of bile acids delivered to the duodenum are recycled via enterohepatic circulation. Bile acids emulsify and facilitate the absorption of dietary lipids. They are also ligands for bile acid receptors, such as transmembrane G protein-coupled receptor 5 (TGR5) and farnesoid X receptor (FXR), which regulate energy and cholesterol metabolism and bile acid transporter gene expression (Wahlstrom *et al.* 2016) (Fig. 2). Deoxycholic acid is associated with hepatocellular carcinoma (Yoshimoto *et al.* 2013) and the inhibition of bacterial overgrowth. Recently, centenarians were found to have a distinct gut microbiome enriched in *Parabacteroides merdae*, *Odoribacter laneus*, and Odoribacteraceae. These microbiota synthesize isoallolithocholic acid, which has antibacterial efficacy against Gram-positive bacteria (Sato *et al.* 2021). Hence, this gut bacteria-derived bile acid might be conducive to human longevity.

Additionally, gut microbiota produces lipids. The genes encoding sphingolipid biosynthesis mediators are distributed mainly in eukaryotes. Nevertheless, certain bacterial species in the Bacteroides phylum also have genes that regulate sphingolipid biosynthesis. *Bacteroides fragilis* produces α-galactosylceramide, which suppresses intestinal natural killer T (NKT) cell activation (An *et al.* 2014). Patients with inflammatory bowel disease (IBD) present with relatively low fecal *Bacteroides* sphingolipid concentrations and are negatively correlated with inflammation. The colonization of germ-free (GF) mice by genetically engineered *Bacteroides thetaiotaomicron*, which lacks sphingolipid synthesis, results in intestinal inflammation and altered host ceramide pools (Brown *et al.* 2019). Thus, sphingolipids produced by *Bacteroides* might maintain intestinal homeostasis.

Gut bacteria also saturate dietary unsaturated fatty acids. *Lactobacillus plantarum* enzymatically metabolizes unsaturated fatty acids via catalysts of hydration and catalysts of dehydration to produce hydroxy-, oxo-, and conjugated fatty acids and partially saturated trans-fatty acids (Kishino *et al.* 2013). Linoleic acid administration activates the arachidonic acid cascade, which induces chronic inflammation in adipose tissue. *Lactobacillus salivarius* and *Lactobacillus gasseri* produce 10-hydroxy-*cis*-12-octadecenoic acid via linoleic acid hydroxylation, which improves obesity, increases GLP-1 secretion, and improves glucose metabolism (Miyamoto *et al.* 2019). Dietary nutrients are major drivers that determine microbial composition. Plasma lipidomic analysis of GF or colonized mice fed different nutrient compositions revealed that the microbiota have dynamic effects on systemic lipid profiles, and the magnitude of this influence is related to metabolic disorders (Watanabe *et al.* 2021). Thus, the gut microbiota comprises a regulator of bile acid metabolism, lipid absorption, inflammatory signals via lipid synthesis, and energy metabolism. Therefore, its disruption can cause metabolic disorders through these mechanisms, which will be discussed in more detail later in this article.

#### Vitamins

Vitamins are either fat soluble (A, D, E, and K) or water soluble (B-complex and C). Fat-soluble vitamins are transported to various tissues and act as signaling molecules and bioactive effectors, whereas water-soluble vitamins act as coenzymes. Vitamin administration alters gut bacteria, increases their biodiversity, and elevates SCFA levels (Pham *et al.* 2021). However, gut bacteria themselves synthesize vitamins (Steinert *et al.* 2020).

Humans cannot synthesize any vitamin, except D. Thus, the human vitamin supply depends on diet and gut bacteria. Gut microbiota produce C, K, and B-complex vitamins. Thiele *et al.* analyzed the microbial genome and assessed the ability of bacteria to synthesize thiamine (B1), riboflavin (B2), niacin (B3), pantothenate (B5), pyridoxine (B6), biotin (B7), folate (B9), and cobalamin (B12). Approximately 40–65% of 256 human commensal bacteria possess a B-complex synthesis pathway (Magnusdottir *et al.* 2015). Interactions occur between vitamins and gut microbiota, and the former affect microbial community structures (Pham *et al.* 2021). Vitamin A administration to neonates within 2 days after birth affects the gut bacterial composition in a sex-dependent manner. It increases *Bifidobacterium* abundance in boys but increases Actinobacteria and *A. muciniphila* abundance in girls (Huda *et al.* 2019). Dietary vitamin A intake and plasma concentrations affect the gut bacterial composition (Wu *et al.* 2011, Li *et al.* 2017), which could aid in protection against various pathogens (Long *et al.* 2007, 2011).

Vitamin D also modifies the gut microbial community structure, and supplementation is associated with significant changes in Firmicutes, Actinobacteria, and Bacteroidetes abundances. Veillonellaceae and Oscillospiraceae (Firmicutes) are negatively associated with 25(OH)-D levels and vitamin D supplementation (Bellerba *et al.* 2021). Vitamin D may protect against IBD by suppressing immunocyte activation, strengthening intestinal barrier functions, and enhancing antibacterial peptide production (Pham *et al.* 2021). Certain bacterial species regulate vitamin D receptors (VDRs). Wu *et al.* reported that the probiotics *Lactobacillus rhamnosus* and *L. plantarum* increase VDR in the IECs and protect against colitis in a VDR signaling-dependent manner (Wu *et al.* 2015). A genome-wide association analysis revealed that variations in VDR affect β-diversity and Parabacteroides abundance (Wang *et al.* 2016). This evidence suggests that gut microbiota regulate vitamin D signaling and help to maintain intestinal homeostasis.

#### Amino acid metabolism

Gut microbiota degrade proteins, produce amino acids (AAs), and metabolize the choline and l-carnitine in red meat to trimethylamine (TMA). The latter enters circulation and is metabolized in the liver to trimethylamine-*N*-oxide (TMAO). In an animal model, TMAO was found to promote atherosclerosis. Plasma TMAO could increase cardiovascular disease (CVD) risk and mortality in a dose-dependent manner (Koeth *et al.* 2013, Tang *et al.* 2013, Schiattarella *et al.* 2017). Wang *et al.* showed that a structural analog of choline, 3,3-dimethyl-1-butanol (DMB), inhibits microbial TMA production. Oral DMB administration reduces TMAO levels and inhibits choline diet-induced macrophage foam cell formation to suppress the development of arteriosclerosis in apolipoprotein E knockout (KO) mice. Therefore, microbial intervention might help to prevent arteriosclerosis (Wang *et al.* 2015).

Gut microbiota also metabolize other AAs. Plasma levels of the branched-chain amino acids (BCAAs) valine, leucine, and isoleucine are correlated with insulin resistance in diabetic patients (Newgard *et al.* 2009, Batch *et al.* 2013, Jang *et al.* 2016). Pedersen *et al.* integrated analyses of serum metabolomics and metagenomics and showed that elevated BCAA levels in insulin-resistant individuals are associated with microbiota, which are enriched in BCAA biosynthesis pathways. *Prevotella copri* and *Bacteroides vulgatus* are the major BCAA synthesizers. The oral administration of *P. copri* to mice fed a high-fat diet results in increased serum BCAAs and aggravates glucose intolerance. Thus, BCAAs produced by gut microbiota could contribute to insulin resistance (Pedersen *et al.* 2016).

The aromatic AAs tyrosine, phenylalanine, and tryptophan are correlated with diabetes risk (Wang *et al.* 2011, Chen *et al.* 2016). Bacteria harbor genes encoding mediators of aromatic AA metabolism. Antibiotic administration strongly affects these metabolic pathways. Hence, aromatic AA metabolism by gut microbiota affects the host AA profile (Fujisaka *et al.* 2018).

### Immune function maintenance and protection against pathogens

SCFAs exert various bioactive effects that help maintain the immune system (Shibata *et al.* 2017). Butyrate is an energy source for IECs and has anti-inflammatory and immunomodulatory effects (den Besten *et al.* 2013). Butyrate interacts with IECs, induces antimicrobial peptide and cytokine production, inhibits pathogen overgrowth, and strengthens intestinal barrier functions (Raqib *et al.* 2006, Singh *et al.* 2014, Shibata *et al.* 2017). The predominant phylum Firmicutes consists mainly of butyrate-producing *Clostridium* clusters IV and XIV (Eckburg *et al.* 2005). Clostridiales bacteria induce regulatory T-cell differentiation in the intestinal mucosa, which helps suppress the immune response (Atarashi *et al.* 2011). Furusawa *et al.* reported that butyrate derived from Clostridiales leads to butyrate-enhanced histone H3 acetylation in both the promoter and conserved non-coding sequence regions of *Foxp3* (Furusawa *et al.* 2013), suggesting that butyrate is an epigenetic modifier for Treg induction.

Bifidobacteria are major producers of acetate, which has immunoregulatory effects. Acetate fortifies the gut barrier function against *Escherichia coli* O157 (Fukuda *et al.* 2011). Acetate induces apoptosis via neutrophil GPR43 and suppresses colitis in a mouse colorectal cancer model (Maslowski *et al.* 2009). Acetate also alleviates allergic airway disease (AAD) in a mouse model. The oral administration of a high-fiber diet or acetate to pregnant mice delays the onset of AAD in their offspring, possibly by inducing Tregs via *Foxp3* acetylation at its promoter and HDAC9 inhibition (Thorburn *et al.* 2015). Succinate, produced by neonate intestinal bacteria, promotes gut microbiota maturation by establishing Clostridiales in the intestinal tract, thereby preventing *Salmonella* and pathogenic *E. coli* growth (Kim *et al.* 2017). Thus, certain bacteria suppress immunocyte activation. In contrast, other bacteria can activate certain immunocytes. Atarashi *et al.* reported that salivary *Klebsiella* spp. ectopically colonizing the intestine activate the Th1 immune response and exacerbate intestinal inflammation in a mouse IBD model (Atarashi *et al.* 2017). In rodents, segmented filamentous bacteria strongly induce Th17cells (Atarashi *et al.* 2015), trigger the production of the proinflammatory cytokines IL-17 and IL-22, and promote antimicrobial peptide production in IECs (Shale *et al.* 2013). In contrast, some bacteria such as* Gordonibacter pamelaeae*, *Eggerthella lenta*, and *Raoultibacter massiliensis* negatively regulate Th17 cell differentiation by producing the secondary bile acid metabolites 3-oxolithocholic acid (3-oxoLCA) and isolithocholic acid (isoLCA). These bile acids inhibit retinoic acid receptor-related orphan nuclear receptor-γ t (RORγt), a key transcription factor that promotes Th17 cell differentiation. Since 3-oxoLCA and isoLCA levels are reduced in patients with IBD and inversely correlated with Th17 cell/IL-17-related genes, decreased 3-oxoLCA and isoLCA in dysbiosis could contribute to IBD pathophysiology (Paik *et al.* 2022). In general, Tregs have anti-inflammatory effects, whereas Th17 cells act in a pro-inflammatory manner in IBD, and the Th17/Treg balance is important to maintain intestinal immune homeostasis, with its dysregulation contributing to IBD development (Ueno *et al.* 2018). Thus, although the mechanisms are complex, it is important to elucidate the interaction between gut commensal bacteria and immunocyte functions.

### Regulation of intestinal motility

Intestinal motility is vital to digestion, absorption, and excretion. Gut bacteria regulate intestinal motility by helping to develop and maintain the intestinal nervous system. Intestinal motility is seldom observed in fetuses with little or no intestinal bacteria (Kien 1996). GF mice present with decreased colonic neuron density and intestinal motility compared with those in specific pathogen-free (SPF) mice. Fecal microbiota transplantation promotes serotonin (5-HT) production in the intestinal mucosa and neurons and increases neuron density and motility (De Vadder *et al.* 2018). Deoxycholic acid produced by spore-forming bacteria enhances 5-HT biosynthesis from colonic enterochromaffin cells and activate platelet functions and intestinal peristalsis (Yano *et al.* 2015). Obata *et al.* compared RNA sequencing of the intestinal tracts of conventionally raised mice with GF mice and found a transcription factor, aryl hydrocarbon receptor (AhR), was induced by microbiota. AhR was expressed in colonic neurons and promoted peristalsis of the intestinal tract, indicating a close link between microbiota and the enteric nervous system (Obata *et al.* 2020). These findings show that intestinal microbiota help to regulate intestinal motility. Therefore, gut dysbiosis can cause chronic constipation and irritable bowel syndrome, which are associated with dysregulated peristalsis. In summary, the gut microbiota play an important role in maintaining systemic homeostasis through the metabolism of nutrients, such as indigestible polysaccharides, lipids, vitamins, and AAs, and regulation of the immune system and intestinal function. In the latter part of this article, we outline the relationship between dysbiosis and obesity/glucose metabolism.

## History of GF animals

Next-generation sequencing technologies such as 16S rRNA sequencing and shotgun metagenomic sequencing have been developed to obtain microbial information. Details of these technologies are described in other reviews (Wensel *et al.* 2022). The transplantation of gut bacteria into GF animals is a powerful tool to investigate the effects of the gut microbiota of interest on biological functions and disease. In this section, we briefly introduce the significance and history of the development of GF animals.

GF animals harbor no detectable microorganisms and can help clarify the roles and significance of the gut microbiota. Gnotobiotic animals are GF and colonized by a single strain or a specific bacterial community comprising various species. They help demonstrate the physiological effects of certain bacteria on the host.

The development of GF animals began in 1885 through the advocacy of Louis Pasteur in France (Pasteur 1885). In 1895, Nuttal and Thierfelder obtained Guinea pigs via aseptic Caesarean section and maintained them in a sterile environment for 13 days (Nuttal & Thierfelder 1985). In 1989, Schottelius *et al.* created the first reported GF chickens. In the early 1900s, Kuster *et al.* established GF goats. Later, GF rats, chickens, and guinea pigs were bred as experimental animals.

GF mice were first weaned in 1954 (Reyniers *et al.* 1946, Reyniers *et al.* 1949, Miyakawa *et al.* 1954). The goal of early GF animal research was to determine whether bacterial symbiosis was beneficial or detrimental to the survival of host organisms. GF animal research is used in physiology, nutrition, bacteriology, and immunology. Current microbiota study tools and techniques have revealed close associations between bacteria and metabolic diseases.

However, maintenance of a gnotobiotic status is expensive and requires experienced staff, and the available facilities for GF animals are limited. Further­more, as GF animals have not innately experienced immunological stimulation by microorganisms, their nutritional status and immune systems are quite different from those of SPF animals. Therefore, we should be cautious in interpreting results of such studies.

## Gut microbiota in obesity

Obesity is one of the most serious and common medical conditions in modern society. According to World Health Organization data, the obese population is increasing annually. In 2016, 39% of all adults (1.9 billion people) were overweight and 13% of all adults (650 million people) were obese. Obesity causes metabolic disorders, hypertension, glucose intolerance, dyslipidemia, and hyperuricemia and is a risk factor for atherosclerosis, ischemic heart disease, and CVD. Therefore, the pathogenesis, prevention, and treatment of obesity merit further investigation. Recent studies have revealed a close relationship between gut microbiota and obesity. The latter alters the gut microbiota, which is in turn closely related to obesity pathogenesis, as it affects host immunity and metabolism. Thus, interventions involving gut microbiota could be used to prevent and treat obesity.

### The gut microbiota is inextricably linked to obesity

Bäckhed *et al.* first found that fecal microbiota transplantation of normal microbiota from the cecum of conventionally raised mice into GF C57/BL6 mice increased fat mass and insulin resistance. The increase in fat storage was not due to an increased food intake but a decreased expression of the fasting-induced adipose factor (Fiaf), a fat accumulation inhibitory factor (Backhed *et al.* 2004). Several years later, they showed the mechanisms by which GF mice are resistant to obesity compared to conventionally raised mice. They found that (1) GF mice had elevated intestinal Fiaf levels, which activated PGC1α in the skeletal muscle, and (2) AMPK in skeletal muscle is activated in GF mice independently of Fiaf signaling. These two phenotypes can lead to enhanced fatty acid oxidation in GF animals, suggesting that the gut microbiota plays an important role in promoting energy accumulation (Backhed *et al.* 2007). Subsequently, several studies showed that the transplantation of gut microbiota from obese human or mouse models into GF mice results in obesity (Turnbaugh *et al.* 2006, Vijay-Kumar *et al.* 2010, Ridaura *et al.* 2013). Furthermore, microbial perturbation via antibiotic administration in childhood has been shown to elevate the risk of obesity in adulthood (Cox *et al.* 2014, Cox & Blaser 2015, Schwartz *et al.* 2016). This led us to recognize that disruption of the microbiota (dysbiosis) is a cause, and not a consequence, of obesity and is closely associated with metabolic diseases (Le Chatelier *et al.* 2013). Thus, the gut microbiota is inextricably linked to obesity.

In 2006, it was first shown that gut bacteria differ with obesity. These findings showed a relative increase in the Firmicutes/Bacteroides (F/B) ratio in obese humans and rodents (Ley *et al.* 2006, Turnbaugh *et al.* 2006). Since then, numerous studies have reported on the gut microbiota of obese patients to clarify this dysbiosis among various ethnic groups and different geographic regions (Table 1). However, the pattern of microbial structure changes with obesity is not constant, and some reports show no change or a decrease in the F/B ratio (Schwiertz *et al.* 2010, Tims *et al.* 2013). Interactions among multiple factors such as race, region, diet, and cultural background might account for these discrepancies. Some human studies have revealed that *Oscillospira* is reduced with obesity (Konikoff & Gophna 2016, Yang *et al.* 2021) and is expected to be a candidate for next-generation probiotics as it produces SCFAs such as butyrate. However, several reports showed that this bacterium is associated with gallstones and chronic constipation, suggesting complex interactions between the bacterium and host physiology (Yang *et al.* 2021). Goodrich *et al.* analyzed the gut microbiota of 416 pairs of twins and found that the abundance of Christensenellaceae was correlated with low BMI and that the transplantation of *Christensenella minuta* into GF mice reduces weight gain (Goodrich *et al.* 2014). Everard *et al.* showed that the relative abundance of *A. muciniphila*, which has been linked to a favorable effect on glucose metabolism, is reduced in obese and diabetic mice and humans (Everard *et al.* 2013). The metabolic ameliorating effects of *A. muciniphila* are described later in the ‘Impaired gut barrier function’ section.

**Table 1:** Characteristics of microbiota associated with obesity among different countries. Characteristics of the microbial composition in obese individuals vary by race and region.

Age	Number	Country	Obese vs normal (excerpted bacteria is shown)		Other phenotype	References
Adults	10 obese/20 lean	Japan	↑*Firmicutes* ↑*Fusobacteria* ↑*Alistipes* ↑*Anaerococcus* ↑*Corpococcus* ↑*Fusobacterium* ↑*Parvimonas*	↓*Bacteroides* ↓*Desulfovibrio* ↓*Faecalibacterium* ↓*Lachnoanaerobaculum* ↓*Olsenella* ↓*Faecalibacterium prausnitzii*	*Bacteroidetes/Firmicutes* (*B/F*) ratio not significant	Andoh *et al.* (2016)
Adults	20 normal weight/20 obese/9 anorexic	France	↑*Lactobacillus* (not significant)	↓*Bacteroidetes*	*Firmicutes* data are similar in the three categories	Armougom *et al.* (2009)
Adults	20 obese/20 normal weight	Italy	↑*Veillonellaceae* ↑*Dialister spp.*	↓*Oscillospira* genus	↓a-diversity	Borgo *et al.* (2013)
Adults	17 obese/25 obese with metabolic syndrome/ 25 healthy	Mexico	↑*Faecalibacterium* ↑*Roseburia* ↑*Lachnospira* ↑*Coprococcus* ↑*Bilophila*	↓*Erysipelotrichaceae*		Chávez-Carbajal *et al.* (2019)
Adults	3 obese/24 overweight/106 normal weight/7 underweight	Italy	↑*Selenomonas* ↑*Megasphaera* ↑*Streptococcus* ↑*Dorea* ↑*Lachnobacterium* ↑*Jannaschia* ↑*Dialister* ↑*Eubacterium*	↓*Paraprevotella*	*Firmicutes/Bacteroidetes* (*F/B*) ratio not significant	Gallè *et al.* (2020)
Adults	24 obese/28 overweight/31 normal weight/21 underweight	Saudi Arabia	↑*Lentisphaerae*			Harakeh *et al.* (2020)
Adults	1674 subjects	USA	↑*Acidaminococcus* ↑*Megasphaera* ↑*Catenibacterium* ↑*Prevotella* ↑*Streptococcus*	↓*Oscillospira* ↓*Cloacibacillus* ↓*Anaerotruncus* ↓*Ruminococcus* ↓*Coprobacillus* ↓*Eggerthella*		Kaplan *et al.* (2019)
Adults	33 obese/23 non-obese	Japan	↑*Blautia hydrogenotorophica* ↑*Coprococcus catus* ↑*Eubacterium ventriosum* ↑*Ruminococcus bromii* ↑*Ruminococcus obeum*	↓*Bacteroides* ↓*Bacteroides faecichinchillae* ↓*Bacteroides thetaiotaomicron* ↓*Blautia wexlerae* ↓*Clostridium bolteae* ↓*Flavonifractor plautii*	↑*F/B* ratio, ↑Shannon–Wiener index	Kasai *et al.* (2015)
Adults	167 normal/396 overweight or obese with different BMI history	Finland	↑*Roseburia* ↑*Blautia*	↓*Rikenellaceae* ↓*Oscillospira*	↓Shannon index	Loftfield *et al.* (2020)
Adults	52 African American/46 Caucasian American	USA			*Bacteroidetes* numbers not significant	Mai *et al.* (2009)
Adults	170 HIV-negative women	South Africa	↑*Prevotella*			Oduaran *et al.* (2020)
Adults	531 subjects (132 obese/100 normal weight)	Finland	↑*Tissierellacea* ↑*Blautia*	↓*Archaea (Methanobrevibacter)*	*F/B* ratio not significant	Org *et al.* (2017)
Adults	248 subjects (83 obese or overweight/83 normal weight/82 underweight)	Bangladesh	↑*Acidaminococcus*	↓*Oscillospira*	↓Chao1 richness, ↓Shannon diversity index	Osborne *et al.* (2020)
Adults	767 subjects	Japan		↓*Alistipes* ↓*Clostridium XlVb* ↓*Erysipelotrichaceae incertae sedis* ↓*Lactobacillus*	*Blautia hansenii* and *Blautia producta* were negatively associated with changes in VFA	Ozato *et al.* (2022)
Adults	1001 subjects	Japan	↑*Prevotella* in men ↑*Clostridium sensu stricto, Roseburia* ↑*Ruminococcus*, and *Megasphaera* in women	↓*Blautia* and *Bifidobacterium* in men ↓*Blautia, Bifidobacterium, Eggerthella, Sutterella*, and *Erysipelotrichaceae incertae sedis* in women	*Blautia* was the only microbiota significantly and negatively associated with VFA, regardless of sex	Ozato *et al.* (2019)
Adults	5 obese/5 surgically treated/5 obese/5 lean	India	↑*Bacteroides*			Patil *et al.* (2012)
Adults	599 subjects ( 142 obese/246 overweight/211 normal weight)	USA	↑*Bacilli* ↑*Streptococcaceae* ↑*Lactobacillaceae*	↓*Clostridia* including *Christensenellaceae* ↓*Clostridiaceae* and *Dehalobacteriaceae*	↓Number of OTUs, ↓Shannon index	Peters *et al.* (2018)
Adults	32 obese/32 normal weight	Mexico	↑*Clostridum leptum* ↑*Lactobacillus*	↓*Prevotella* ↓*Escherichia coli*		Radilla-Vazquez *et al.* (2016)
Adults	60 subjects (25 obese with T2D/25 obese non-diabetic/5 non-obese with T2D/5 control)	Egypt	↑*Prevotella* ↑*Clostridium* ↑*Faecalibacterium* ↑*Staphylococcus*	↓*Akkermansia*	↑*F/B* ratio	Salah *et al.* (2019)
Adults	33 obese/35 overweight/30 normal weight	Germany	↑*Bacteroidetes*	↓*Bifidobacterium* ↓*Methanobrevibacter spp.* ↓*Ruminococcus flavefaciens* subgroup	↓*F/B* ratio	Schwiertz *et al.* (2010)
Adults	20 twin pairs	Finland			The abundance and diversity of the bacterial groups not significant	Simoes *et al.* (2013)
Adults	1280 subjects (633 lean non-diabetic/494 obese non-diabetic/153 obese with T2D)	Germany	↑*Bacteroides thetaiotaomicron*	↓*Faecalibacterium prausnitzii*		Thingholm *et al.* (2019)
Adults	20 concordant/20 discordant BMI twin pairs	Netherlands	↑*Eubacterium ventriosum*, ↑*Roseburia intestinalis*	↓*Oscillospiraguillermondii*	*B/F* ratio not significant	Tims *et al.* (2013)
Adults	52 obese/52 normal weight	China		↓*Clostridium perfringens*, ↓*Bacteroides*		Zuo *et al.* (2011)
Children	15 obese/13 normal weight	India	↑*Fecalibacterium prausntzi*			Balamurugan *et al.* (2010)
Children	25 overweight/7 obese/24 normal weight	Finland	↑*Staphylococcus aureus*	↓*Bifidobacteria*		Kalliomaki *et al.* (2008)
Children	15 obese/15 normal weight	Swiss			No significant quantitative differences	Payne *et al.* (2011)
Children	138 subjects	Flanders	↑*Bacteroides fragilis*	↓*Staphylococcus*		Vael *et al.* (2011)

More recently, metabolomics has been used to elucidate the effects of metabolites produced by the gut microbiota. In China, a metagenome-wide association analysis and serum metabolomic profiling revealed that the abundance of glutamate-fermenting *B. thetaiotaomicron* is reduced with obesity and associated with an elevation in serum glutamine (Gln). Bariatric surgery and *B. thetaiotaomicron* administration decrease Gln levels in mice. Hence, *B. thetaiotaomicron* affects AA cycling and has anti-obesity effects (Liu *et al.* 2017). In contrast, other researchers reported that *B. thetaiotaomicron* upregulates intestinal lipid absorption transporters, promotes hepatic lipid biosynthesis, and causes obesity and impaired glucose tolerance in high-fat diet-fed mice (Cho *et al.* 2022). Further research is required to identify the mechanism through which gut bacteria help to improve obesity and metabolic disorders.

### Obesity treatment alters the gut microbiota

Bariatric surgery is a well-established obesity treatment. It promotes weight loss and improves obesity-related metabolic disorders such as hypertension, diabetes, and dyslipidemia (Courcoulas *et al.* 2018) by reducing food intake and altering gut hormone and bile acid levels, energy metabolism, and the gut microbiota (Debedat *et al.* 2019). Several studies have shown that the microbial community structure is altered after bariatric surgery. Nevertheless, the results and conclusions were inconsistent among reports possibly because of differences in study designs, sample sizes, subject races, geography, and diet (Table 2). Ryan *et al.* demonstrated that the metabolic effects of sleeve gastrectomy are abolished in FXR-disrupted mice, suggesting that FXR signaling contributes to body weight reduction and improvement in glycemic control after bariatric surgery (Ryan *et al.* 2014). Another group elucidated the mechanism by which Roux-en-Y gastric bypass (RYBG) improves metabolism in rodents. Specifically, it decreases bile acids and increases glucagon-like peptide 1 (GLP-1) via L-cell proliferation. The decrease in bile acids, associated with a decline in *Lactobacillus* abundance, results in intestinal L-cell proliferation, an increase in GLP-1 secretory capacity, and improvements in glycemic control (Dang *et al.* 2021). Torsten *et al.* analyzed 40 pre-RYBG and post-RYBG cases and showed that RYBG improved chronic inflammation but increased proinflammatory Proteobacteria. Lipopolysaccharide (LPS) and flagellin-specific immunoglobulin A (IgA) were increased, but the total fecal IgA content remained unchanged. Hence, an increase in intestinal IgA after bariatric surgery neutralized immunogenic bacteria and their components, leading to the improved systemic inflammation (Scheithauer *et al.* 2022). Thus, metabolic improvements mediated by bariatric surgery are mainly associated with changes in incretin secretion and immune activities that are mediated by microbial alterations.

**Table 2:** Characteristics of microbiota after bariatric surgery in different countries.

Operation	Number	Country	Major bacterial changes after surgery		Diversity after surgery	References
Roux-en-Y gastric bypass	14	Brazil		↓F/B ratio	↑OTUs richness	Al Assal *et al.* (2020)
Gastric banding or Roux-en-Y gastric bypass	24	France	↑*Butyricimonas virosa* , 11 altered after Roux-en-Y and 2 altered after gastric banding		↑Microbial gene richness	Aron-Wisnewsky *et al.* (2019)
Roux-en-Y gastric bypass or sleeve gastrectomy	53	China	33 altered after sleeve and 19 altered after Roux-en-Y		↑Richness and evenness	Chen *et al.* (2020)
Roux-en-Y gastric bypass	24	China	↑*Bacteroidetes* ↑*Bifidobacterium*			Chen *et al.* (2017)
Roux-en-Y gastric bypass or sleeve gastrectomy	197	France, Switzerland, USA	↑*Akkermansia muciniphila*		↑Shannon index and gene richness	Farin *et al.* (2020)
Sleeve gastrectomy	10	Japan	↑*Bacteroidetes* ↑*Fusobacteria*		↑Faith PD ↑Chao1 ↑Shannon index	Fukuda *et al.* (2022)
Roux-en-Y gastric bypass	30	France	↑*Bacteroides/Prevotella* ↑*E. coli*	↓*Bifidobacterium/ Lactobacillus/ Leuconostoc* ↓*Pediococcus*		Furet *et al.* (2010)
Roux-en-Y gastric bypass or sleeve gastrectomy	31	Korea	↑*Streptococcus* ↑*Oscillospira* ↑*Akkermansia*	↓*Prevotella* ↓*Turicibacter* ↓*Bifidobacterium*	↑Observed species	Han *et al.* (2022)
Sleeve gastrectomy or sleeve with duodenojejunal bypass or gastric banding	44	Japan	↑*Bacteroidetes* ↑*Lactobacillales* ↑*Enterobacteriales*			Kikuchi *et al.* (2018)
Roux-en-Y gastric bypass	30	France	↑*Proteobacteria* ↑*Bacteroides* ↑*Escherichia*	↓*Lactobacillus* ↓*Dorea* ↓*Blautia* ↓*Bifidobacterium*	↑Richness	Kong *et al.* (2013)
Sleeve gastrectomy	23	China			↑α-diversity	Liu *et al.* (2017)
Roux-en-Y gastric bypass or sleeve gastrectomy	28	Spain	(Sleeve) ↑*Akkermansia* ↑*Haemophilus* (Roux-en-Y) ↑*Clostridium* ↑*Fusobacterium*	(Sleeve) ↓*Anaerostip* ↓*Bifidobacterium* (Roux-en-Y) ↓*Bifidobacterium* ↓*Collinsella*		Sanchez-Alcoholado *et al.* (2019)
Roux-en-Y gastric bypass or sleeve gastrectomy	45	Poland	↑*Bacteroidetes* ↑*Bacteroidales* ↑*Bacteroidia* ↑*Prevotellaceae* ↑*Rikenellaceae*	↓*Firmicutes* ↓*Clostridiales* ↓*Clostridia* ↓*Lachnospiraceae* ↓*Blautia*		Stefura *et al.* (2022)
Roux-en-Y gastric bypass	40	Netherlands	↑*Proteobacteria* ↑*Akkermansia*	↓*Roseburia* ↓*Bacteroides* ↓*Faecallibacterium*	↑α-diversity	Scheithauer *et al.* (2022)

## Gut microbiota and glucose metabolism

Research on the relationships among gut microbiota, diabetes, and obesity has progressed. Gut microbiota differ by nationality and race in patients with type 2 diabetes (T2DM). A metagenomic analysis of a Chinese T2DM cohort conducted by Qin *et al.* revealed decreased butyrate-producing bacteria, increased methane metabolism and hydrogen sulfide production, and upregulation of the expression of genes regulating oxidative stress resistance (Qin *et al.* 2012). A shotgun metagenomic analysis conducted by Karlsson *et al.* on a cohort of 145 European women with a normal status, impaired glucose tolerance, and T2DM characterized compositional and functional alterations in the gut microbiota. They established a random forest model based on the metagenomic data that could be applied to patients with impaired glucose tolerance. However, this model could not be consistently applied to the cohort of Qin *et al.* (Karlsson *et al.* 2013). Hence, metagenomic profiles differ between diabetic and healthy individuals and can be a good predictive tool for impaired glucose metabolism, but regional and racial differences in gut microbiota must be considered. There is nonetheless a strong link between microbial dysfunction and impaired glucose metabolism. Moreover, gut dysbiosis is already present at the impaired glucose tolerance stage, even before T2DM onset. A metagenomic analysis conducted by Wu *et al.* on ~1500 Swedish subjects showed that prediabetes and T2DM are characterized by alterations in the gut microbiota and their functional genes and decreases in the abundance of butyrate-producing bacteria. The authors constructed a machine learning model using a random forest algorithm to distinguish individuals with prediabetes or diabetes and showed that bacterial compositional/functional alterations are associated with insulin resistance at the impaired glucose tolerance stage (Wu *et al.* 2020). Gut microbiota analysis could help diagnose early-stage glucose intolerance and improve early intervention. Based on these studies, the construction of a machine learning model to predict the development of T2DM in healthy and prediabetic patients has been challenging (Aasmets *et al.* 2021). However, it could serve as a predictive marker for individual risks of metabolic disorders.

Gut microbiota are also implicated in type 1 diabetes (T1DM). Gavin *et al.* proteomically analyzed stool samples of patients with T1DM and detected proteins associated with intestinal inflammation, reduced intestinal barrier functions, and changes in gut microbiota even before disease onset (Gavin *et al.* 2018). Tracking the gut microbiota of children congenitally predisposed to T1DM revealed reduced microbial diversity and transient spikes in *Ruminococcus gnavus* and *Streptococcus infantarius* abundance before disease onset (Kostic *et al.* 2015). A metagenomic analysis of 74 T1DM patients and 296 healthy controls showed microbial differences between groups; *P. copri* and *Eubacterium siraeum* were more common in T1DM, whereas Firmicutes and *Faecalibacterium prausnitzii* were more prevalent in controls. In this study, altered bacterial species and metabolic pathways were associated with host glycemic control (Shilo *et al.* 2022). Thus, changes in gut microbiota and the ambient environment might be associated with T1DM development. As an immunological mechanism, the commensal gut bacterium *Parabacteroides distasonis* has an epitope similar to the insulin beta chain, the target protein of the autoimmune response in T1DM. The presence of this bacteria could cause the production of autoimmune antibodies leading to the pathogenesis of T1DM (Girdhar *et al.* 2022). However, some reports suggest that the microbiota inhibit the development of T1DM. MyD88-KO NOD mice develop T1DM in a GF environment but not in the presence of intestinal bacteria (Wen *et al.* 2008). Shimokawa *et al.* reported that *Ruminococcus* spp. might prevent the onset of T1DM by inducing CD8+ regulatory T cells (Shimokawa *et al.* 2020), suggesting a close interaction between the microbiota and immune system and the onset of T1DM. This evidence clearly implicates the involvement of microbiota in glucose metabolism. The mechanisms by which microbiota contribute to glucose metabolism are described in the following sections.

### Short-chain fatty acids

SCFAs exert their metabolic effects through G protein-coupled fatty acid receptors such as GPR41 and GPR43. GPRs are involved in GLP-1 and peptide YY (PYY) secretion in intestinal L cells (Samuel *et al.* 2008, Tolhurst *et al.* 2012) to regulate insulin secretion by stimulating pancreatic β-cells and energy balance (Natarajan & Pluznick 2014, McNelis *et al.* 2015). Mice lacking these receptors exhibit reduced GLP-1 and PYY secretion after SCFA administration (Psichas *et al.* 2015, Koh *et al.* 2016, Brooks *et al.* 2017). Butyrate enhances thermogenesis in skeletal muscle and brown adipose tissue (BAT), improves glucose metabolism by upregulating PGC-1α, AMPK, and p38 expression (Gao *et al.* 2009), promotes energy expenditure via GPR41 in sympathetic ganglia, and contributes to *in vivo* energy homeostasis (Inoue *et al.* 2012). Kimura *et al.* also reported that SCFAs regulate energy metabolism by promoting sympathetic activation via GPR41 signaling (Kimura *et al.* 2011). SCFA-mediated GPR43 activation in adipocytes inhibits insulin signaling by suppressing Akt phosphorylation to reduce fat accumulation (Kimura *et al.* 2013). This evidence suggests that increasing SCFAs in the body might be metabolically beneficial. In addition to the intake of non-digestible polysaccharides, physical activity (Magzal *et al.* 2022) and cold stimuli (Ichikawa *et al.* 2021) are implicated in SCFA production. Prior attempts have been made to ameliorate metabolic diseases using SCFA-related interventions. Emanuel *et al.* reported that infusions of SCFAs, such as acetate, butyrate, and propionate, into the distal colon increase fasting fat oxidation, resting energy expenditure, and PYY concentrations in men (Canfora *et al.* 2017).

### FXR/TGR5/bile acids

FXR (NR1H4) is a member of the nuclear receptor superfamily of TFs that senses and regulates bile acid, lipid, and glucose metabolism (Matsubara *et al.* 2013, Sayin *et al.* 2013). Gut microbiota regulate FXR signaling by reducing the FXR antagonist muricholic acid (Sayin *et al.* 2013). Mice lacking FXR signaling exhibit dyslipidemia (Sinal *et al.* 2000). The FXR agonists 6-ethyl-chenodeoxycholic acid, fexaramine, and GW 4064 and FXR overexpression improve the metabolic profile in a mouse obesity model (Zhang *et al.* 2006, Cipriani *et al.* 2010, Fang *et al.* 2015). In contrast, FXR-KO mice display improvements in metabolic disturbances induced by a high-fat diet (Prawitt *et al.* 2011) and increased GLP-1 secretion (Trabelsi *et al.* 2015). Jiang *et al.* reported that oral administration of the selective high-affinity FXR inhibitor glycine-β-muricholic acid ameliorates obesity, insulin resistance, and fatty liver (Jiang *et al.* 2015). These conflicting results suggest that FXR plays complex roles in the etiology of metabolic dysfunction and that its effects on metabolism vary in an organ-dependent manner. Future research should aim to clarify the significance of FXR in glucose metabolism.

TGR5 is a membrane-bound or G-protein bile acid-activated receptor (Kawamata *et al.* 2003). *TGR5* expression is ubiquitous in human and rodent tissues and is upregulated in the lungs, liver, gallbladder, spleen, adipose tissue, CNS, and intestine (Duboc *et al.* 2014). Bile acids stimulate the secretion of intestinal hormones that regulate blood glucose and appetite (Adrian *et al.* 2012), such as GLP-1 and PYY. The latter is a centrally acting appetite suppressant (Kuhre *et al.* 2018). Bile acids also affect metabolism via TGR5 receptors in BAT. In mice, bile acid-induced TGR5 activation in BAT increases energy expenditure by inducing the cAMP-dependent thyroid hormone-activating enzyme known as type 2 iodothyronine deiodinase (D2), leading to improved obesity tolerance and metabolic disease. This increase in TGR5-mediated energy expenditure is abolished in D2^−/−^ mice. The authors also confirmed that bile acid induces D2 expression and elevated oxygen consumption in a TGR-dependent fashion in human skeletal muscle cells. Hence, bile acid-TGR5-cAMP-D2 signaling might be protective against obesity in humans (Watanabe *et al.* 2006).

*In vitro*, bile acids could decrease endoplasmic reticulum (ER) stress, which is increased in obesity and closely related to insulin resistance etiology. ER stress blocks insulin signaling by overactivating c-Jun N-terminal kinase and promoting the serine phosphorylation of insulin receptor substrate-1 (Ozcan *et al.* 2004), inducing β-cell dysfunction and diabetes onset. Ozcan *et al.* reported that taurine-bound ursodeoxycholic acid reduces ER stress in cultured cells and whole animals. The administration of this conjugated bile acid to obese and diabetic mice normalizes hyperglycemia, restores systemic insulin sensitivity, improves fatty liver, and enhances insulin actions in liver, muscle, and adipose tissues (Ozcan *et al.* 2006). Alterations to the bile acid profile through microbiota modification have been investigated for the improvement of metabolic disorders. Jielong *et al.* reported that phenolic blueberry extracts increase energy expenditure in BAT and improve hepatic lipid metabolism via TGR5 and FXR. These effects are strongly correlated with bile acid regulation, reduction of the FXR inhibitors TαMCA and TβMCA, and expansion of the gut microbiota, including *Bifidobacterium* spp. and *Lactobacillus* spp. Antibiotic administration attenuates the aforementioned metabolic effects (Guo *et al.* 2019).

Chronic antibiotic treatments such as vancomycin or metronidazole change bile acid composition due to a loss of intestinal bacteria harboring bile acid-converting enzymes and resulted in the inhibition of inflammatory secondary bile acids production. Furthermore, the changes in the bile acid composition induced anti-inflammatory TGR5 in the liver. As a result, antibiotic treatment mitigated high-fat diet-induced systemic inflammation in C57BL6 mice. However, these effects were not observed in 129S1 or 129S6 mice. Hence, antibiotic modification of the gut microbiota and changes in bile acid and inflammatory signaling can improve glucose metabolism. However, these effects vary by host genetic background and inflammatory potential (Fujisaka *et al.* 2016).

### Imidazole propionate

Imidazole propionate (ImP) is a histidine (His) metabolite produced by the gut microbiota. A study on 1990 subjects in 3 European countries showed elevated ImP levels in the serum of prediabetic and T2DM patients. Nevertheless, there was no correlation between His consumption and ImP levels, suggesting that the gut microbiota is largely responsible for increases in ImP (Molinaro *et al.* 2020). ImP exacerbates insulin resistance by activating the mTOR pathway and inhibiting insulin signaling (Koh *et al.* 2018). Thus, ImP might contribute to metabolic disease progression. *Clostridium bolteae*, *Cenarchaeum symbiosum*, and *R. gnavus* might affect ImP levels. High saturated fatty acid, low fiber, and low unsaturated fatty acid diets are associated with elevated ImP. Furthermore, ImP levels are high in patients with poorly controlled T2DM, and ImP inhibits the action of metformin. The glucose-lowering effect of metformin is abolished in mice pretreated with ImP, via the inhibition of AMPK phosphorylation in a p38γ-dependent manner (Koh *et al.* 2020). Thus, metabolites produced from AAs by gut microbiota not only modulate insulin signaling but also influence drug action.

### Impaired gut barrier function

Dysbiosis causes so-called ‘leaky gut’ syndrome wherein the intestinal barrier function is impaired, with increased permeability, via decreases in intestinal mucus and tight junction protein content in the intestinal epithelium. With increased intestinal permeability, the bacterial cell wall component endotoxin enters circulation and causes hyper-endotoxemia. Endotoxin activates Toll-like receptor (TLR) 4, which in turn promotes chronic inflammation in the adipose tissue and liver and exacerbates obesity-induced insulin resistance (Amar *et al.* 2008, Gummesson *et al.* 2011, Lassenius *et al.* 2011). Impaired barrier functions can be treated by microbial intervention. Amer *et al.* found that administration of the probiotic *Bifidobacterium animalis* prevents LPS invasion into circulation and improves the inflammatory and metabolic status of diet-induced obese mice (Amar *et al.* 2011). In humans, supplementation with the probiotics *Bacillus indicus*, *Bacillus subtilis*, *Bacillus coagulans*, *Bacillus licheniformis*, and *Bacillus clausii* reduces serum endotoxin levels by 42% (McFarlin *et al.* 2017). Thus, intervention with gut bacteria, to strengthen gut barrier functions, is a novel therapeutic approach.

*A. muciniphila* is an oval, nonmotile, Gram-negative bacterium of phylum Verrucomicrobia. In 2004, Derrien isolated it from healthy human feces inoculated on media containing mucin as the nutrient source (Derrien *et al.* 2004). The genus *Akkermansia* (of *A. muciniphila*) is derived from that of the Dutch microbial ecologist Antoon Akkermans. In both obese humans and rodents, reductions in *A. muciniphila* abundance have been reported (Everard *et al.* 2013, Le Chatelier *et al.* 2013). By contrast, therapeutic interventions that increase *A. muciniphila* are associated with improvements in obesity or glucose metabolism. For example, when mice fed a high-fat, high-sucrose diet are treated with polyphenol-rich cranberry extract, the abundance of *A. muciniphila* is increased, which is associated with improved obesity, insulin resistance, and intestinal inflammation (Anhe *et al.* 2015). Bofutsushosan is a Chinese herbal medicine that improves insulin resistance by increasing *A. muciniphila* and improving intestinal barrier functions (Fujisaka *et al.* 2020). Metformin also modulates the gut microbiota, promotes *A. muciniphila* growth, and improves glucose metabolism (Shin *et al.* 2014, de la Cuesta-Zuluaga *et al.* 2017). Prebiotics such as isomalto-oligosaccharides and red pitaya betacyanin significantly increase *A. muciniphila* and improve glucose metabolism (Song *et al.* 2016, Singh *et al.* 2017). *A. muciniphila* helps maintain intestinal and mucosal epithelial cell functions and suppresses intestinal inflammation leading to improved metabolism in diabetic and obese patients (Everard *et al.* 2013, Plovier *et al.* 2017, Ansaldo *et al.* 2019). Crala *et al.* reported that pasteurized *A. muciniphila* administration decreases body weight in a mouse diet-induced obesity model by increasing systemic energy expenditure and fecal energy excretion (Depommier *et al.* 2020). In a randomized, double-blind, controlled trial on *A. muciniphila* in humans, *A. muciniphila* transplantation improved weight loss, total cholesterol levels, and insulin resistance compared to those with a placebo. Furthermore, pasteurized *A. muciniphila* had higher efficacy than live* A. muciniphila*. Hence, *A. muciniphila* administration might be both a safe and effective treatment (Plovier *et al.* 2017, Depommier *et al.* 2019). Kim *et al.* demonstrated that whereas live *A. muciniphila* suppresses diet-induced fatty liver (Kim *et al.* 2020), pasteurized *A. muciniphila* improves intestinal barrier functions but does not impede the progression of nonalcoholic steatohepatitis (Morrison *et al.* 2022). These results suggest that live and pasteurized* A. muciniphila* are beneficial for obesity-related metabolic disorders but have different effects on the host, and further research is required to clarify them.

## Gut microbiota and hepatic diseases

The liver receives >70% of its blood flow from the portal vein and is continuously exposed to nutrients and bacteria-related substances from the intestine. Therefore, compositional and functional changes in microbiota play important roles in hepatic disease pathogenesis. A healthy intestinal barrier maintains its function and prevents gut microbiota from reaching the liver. However, when the barrier is impaired by various environmental factors, such as an unbalanced diet, gut bacteria and/or their components can be translocated to the liver. TLRs and pathogen recognition receptors, such as the inflammasome, recognize bacterial antigens and enhance the production of proinflammatory cytokines by activating hepatic macrophages and the immune response (Regnier *et al.* 2021). Dysbiosis is associated with the pathological conditions of various hepatic diseases such as alcoholic hepatitis, nonalcoholic fatty liver disease (NAFLD), cirrhosis, and hepatocellular carcinoma (HCC). Chronic alcohol intake has been reported to cause dysbiosis and impair intestinal barrier functions in animals and humans (Yan *et al.* 2011, Mutlu *et al.* 2012). *Enterococcus faecalis* is increased in alcoholic hepatitis patients and secretes cytolysin, which causes hepatocyte death and liver injury. In one study, bacteriophages targeting cytolysin-producing *E. faecalis* abolished ethanol-induced liver damage (Duan *et al.* 2019). Future research should establish whether this approach improves alcoholic hepatitis in humans.

Loomba *et al.* assessed hepatic fibrosis severity based on biopsies of 86 American patients with NAFLD. Whole-genome shotgun sequencing of stool samples revealed that advanced hepatic fibrosis is associated with increased Proteobacteria and decreased Firmicutes, *Ruminococcus obeum*, and *Eubacterium rectale* abundance (Loomba *et al.* 2017). In contrast, Boursier *et al.* showed that the relative abundances of Bacteroides and *Ruminococcus* were increased and that of *Prevotella* was decreased in French patients with advanced hepatic fibrosis (Boursier *et al.* 2016). Race, geographical factors, diet, and patient enrollment criteria also substantially influence gut microbial responses and must be considered. However, changes in gut microbiota can cause hepatic fibrosis. In the future, gut microbiota could be used to predict the risk of hepatic diseases.

Gut microbiota are also implicated in the development of HCC. Deoxycholic acid is increased in obesity-induced gut dysbiosis, induces the senescence-associated secretory phenotype in hepatic stellate cells, and is associated with various proinflammatory and tumor-promoting factors in the liver. These processes promote HCC development in mice exposed to chemical carcinogens (Yoshimoto *et al.* 2013). Ma *et al.* demonstrated that hepatic CXCR-positive NKT cells have antitumor efficacy in mouse spontaneous HCC and metastatic liver cancer models. Primary bile acids upregulate, whereas bacteria-derived secondary bile acids downregulate, CXCL16 expression, which in turn induces hepatic NKT cells. *Clostridium scindens* produces secondary bile acids that inhibit hepatic NKT cells (Ma *et al.* 2018). These studies demonstrate that the gut microbiota is vital in regulating hepatocarcinogenesis through bile acid signaling.

## Conclusions

**Figure 3:**
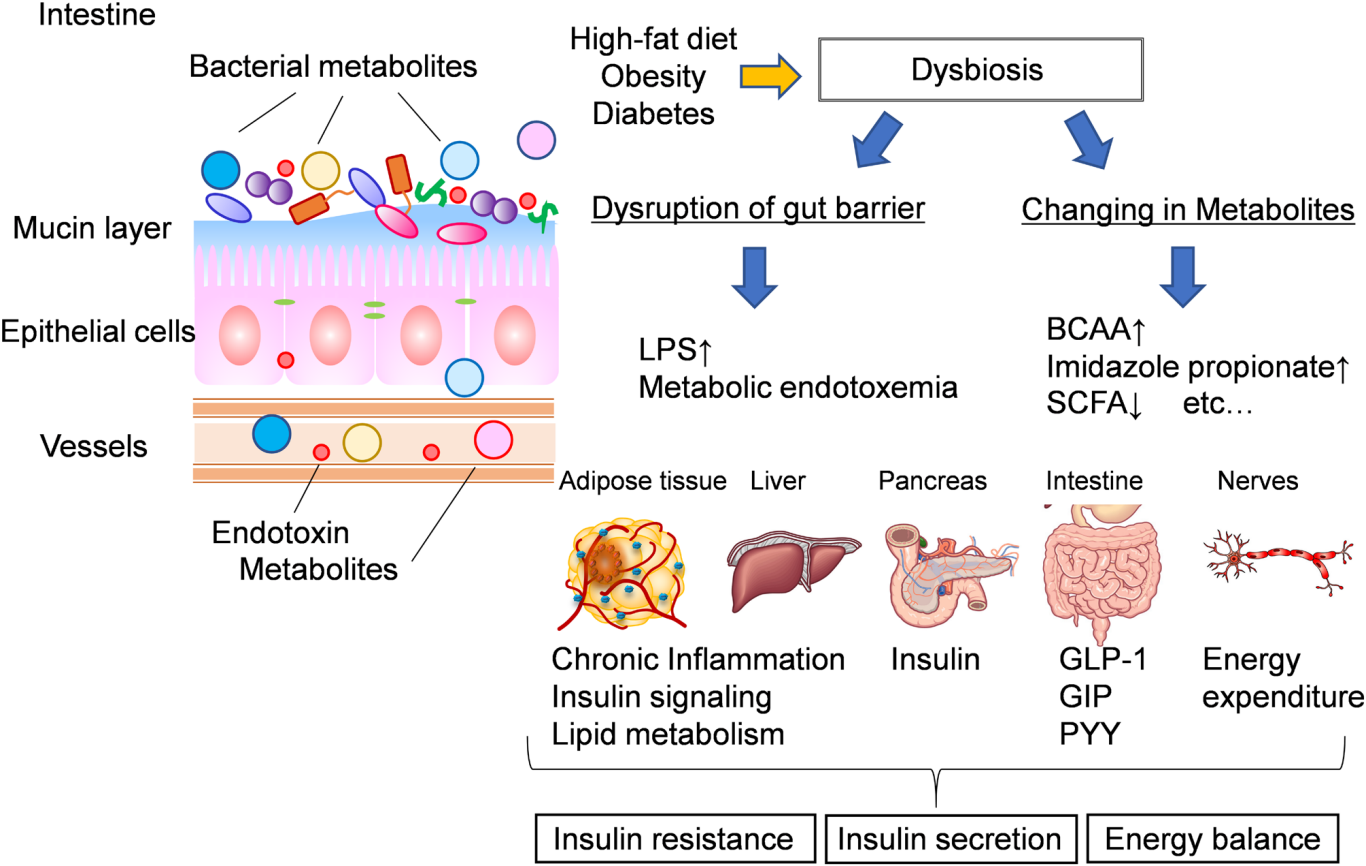
Mechanisms of dysbiosis-induced impaired glucose metabolism. There are two major mechanisms of impaired glucose metabolism mediated by dysbiosis. One is the disruption of the intestinal barrier function, which causes LPS to enter circulation, inducing chronic inflammation and exacerbating insulin resistance, and the other is the effect of microbial metabolites. Increased branched-chain amino acids (BCAAs), imidazole propionate, and decreased short-chain fatty acids, such as butyrate, can affect insulin resistance in various organs, insulin secretion, and energy expenditure.

In recent years, as our understanding of the physiological role of the gut microbiota has advanced, the mechanisms by which its dysfunction can cause metabolic disorders have gained clarity (Fig. 3). Future research should aim to elucidate the complex roles of the gut microbiota in these processes. These discoveries might lead to the application of the gut microbiota and their metabolites for the future treatment of metabolic diseases.

## Declaration of interest

K.T. received lecture fees/grants from Mitsubishi Tanabe Pharma Corporation., MSD K.K., Novo Nordisk Pharma Ltd., Daiichi Sankyo Co. Ltd., Takeda Pharmaceutical Co. Ltd., Suntory Global Innovation Center Ltd., Mitsubishi Tanabe Pharma Corporation, Asahi Kasei Pharma Corporation, and the Mitsubishi Foundation.

## Funding

Our lab is supported by grants from the JSPS (Japan Society for the Promotion of Science), KAKENHI grant numbers 17K09821 and 20K08882 to S.F., grants from AMED PRIME (JP18gm6010023h0001) to S.F., grants from AMED-FORCE (JP21gm4010014h0001) to K.T., and grants from the Japan Diabetes Foundation, Research Funding Granted by the President of the University of Toyama, Research Funding Granted by the Japan Society for the Study of Obesity (JASSO) and Novo Nordisk Pharma Ltd. and Takeda Science Foundation to S.F. Y.W. received grants from the JSPS (Japan Society for the Promotion of Science) KAKENHI grant numbers 21K20896 and 22K16424, grants from the Lotte Foundation, and the Yakult Bio-Science Foundation.
